# Fibromyalgia in the workplace: risk factors for sick leave are related to professional context rather than fibromyalgia characteristics— a French national survey of 955 patients

**DOI:** 10.1186/s41927-019-0089-0

**Published:** 2019-10-26

**Authors:** F. Laroche, D. Azoulay, A. P. Trouvin, J. Coste, S. Perrot

**Affiliations:** 1 Pain department, Paris Medicine Sorbonne University and Saint-Antoine Hospital, 184 rue du Faubourg Saint Antoine, 75012 Paris, France; 2 Pain department, Cochin Hospital, Paris Descartes University, INSERM U987, 27, rue du Faubourg Saint-Jacques, 75014 Paris, France; 3 Biostatistics, Cochin Hospital, Paris Descartes University, Paris, France

**Keywords:** Fibromyalgia, Online survey, Workplace, Sick leave

## Abstract

**Background:**

Work and workplace factors are important in fibromyalgia management. We investigated factors associated with sick leave in professionally active women living with fibromyalgia.

**Methods:**

A questionnaire for fibromyalgia patients in employment was developed by pain and occupational physicians and patients’ organizations. Women in full-time work, screened for fibromyalgia with the FiRST questionnaire, were recruited for a national online survey. Sick leave over the preceding year was analyzed.

**Results:**

In 5 months, we recruited 955 women, with a mean of 37 days of sick leave in the previous year: no sick leave (36%), up to 1 month (38%), 1 to 2 months (14%), more than 2 months (12%). In the groups displayed no differences in demographic characteristics, fibromyalgia symptoms, functional severity and psychological distress were observed. However, they differed in workplace characteristics, commute time, stress and difficulties at work, repetitive work, noisy conditions, career progression problems and lack of recognition, which were strong independent risk factors for longer sick leave. Sedentary positions, an extended sitting position, heavy loads, exposure to thermal disturbances and the use of vibrating tools did not increase the risk of sick leave.

**Conclusions:**

Women with fibromyalgia frequently take sick leave, the risk factors for which are related to the workplace rather than fibromyalgia characteristics.

**Perspective:**

This is the first study to assess the impact of occupational and clinical factors on sick leave in women living with fibromyalgia. Risk factors were found to be related to the workplace rather than fibromyalgia and personal characteristics. Workplace interventions should be developed for women with fibromyalgia.

## Introduction

Fibromyalgia (FM) is a frequent diffuse chronic pain disorder with an estimated prevalence between 1.5 and 4% [1]. It is much more frequent among women than men (sex ratio of 2:1) according to the 2016 revised diagnostic criteria [2], and is considered to be the second most important rheumatologic disorder after osteoarthritis [3].

Fibromyalgia impacts the professional sphere, with significant economic consequences for the patients, their employers, and society [3]. Winkelmann et al. estimated the economic and financial burden of FM in Europe in 2011 [4] at a total annual cost per person of €7900, consisting of €910 of direct costs and €6990 of indirect costs. In total, 88.5% of fibromyalgia-related costs were attributable to a loss of productivity and disability [4]. Employment rates for people suffering from FM vary, ranging from 34 to 77% [5–7].

Work effects are not always deleterious, as employment has been shown to protect against pain and fatigue [5, 6, 8, 9]. A US study of 287 women with fibromyalgia performed in 2003 found that those who worked were better off than those who did not [10]. Work also provides financial stability and better social support [11]. However, the symptoms of the disease can impair work capacity, limit career progression and cause misunderstandings between patients, colleagues and employers [12–14].

People with FM have higher risks of unemployment and long-term disability than the general population [5, 12]. Disability rates vary between 25 and 50% [13, 15]. In a qualitative study of 39 FM patients, women included expressed problems with usual work schedules, repeating actions, decrease in productivity and concentration problems [13]. These difficulties may make it difficult for patients to do their jobs and may lead to repeated absences [13]. Employees suffering from FM have been reported to take three times more sick leave than other workers without FM [16].

We therefore investigated factors associated with sick leave during the previous 12 months in professionally active women living with FM.

## Methods

### Study design

We performed a cross-sectional descriptive study in France, in 2014, on a population of women reporting fibromyalgia. We used data collected in a national online survey, to which 4516 individuals responded [17]. This survey, described previously, was performed by the SOS Fibromyalgia Association [17]. It was developed with the assistance of three medical experts (rheumatologists and pain specialists). The questionnaire was built to explore all domains of fibromyalgia, described by the Outcome Measures in Rheumatology (OMERACT) initiative on fibromyalgia, on a e-health platform already developed and tested in rheumatoid arthritis: the Sanoia platform [18]. An initial version of the questionnaire was tested on patients and was then adapted to obtain the definitive version. Patients visiting the website of the association were asked to complete the questionnaire on the French Sanoia platform “to establish an inventory of the principal repercussions, needs and expectations to improve their everyday life.”

### Population

**Fig. 1:**
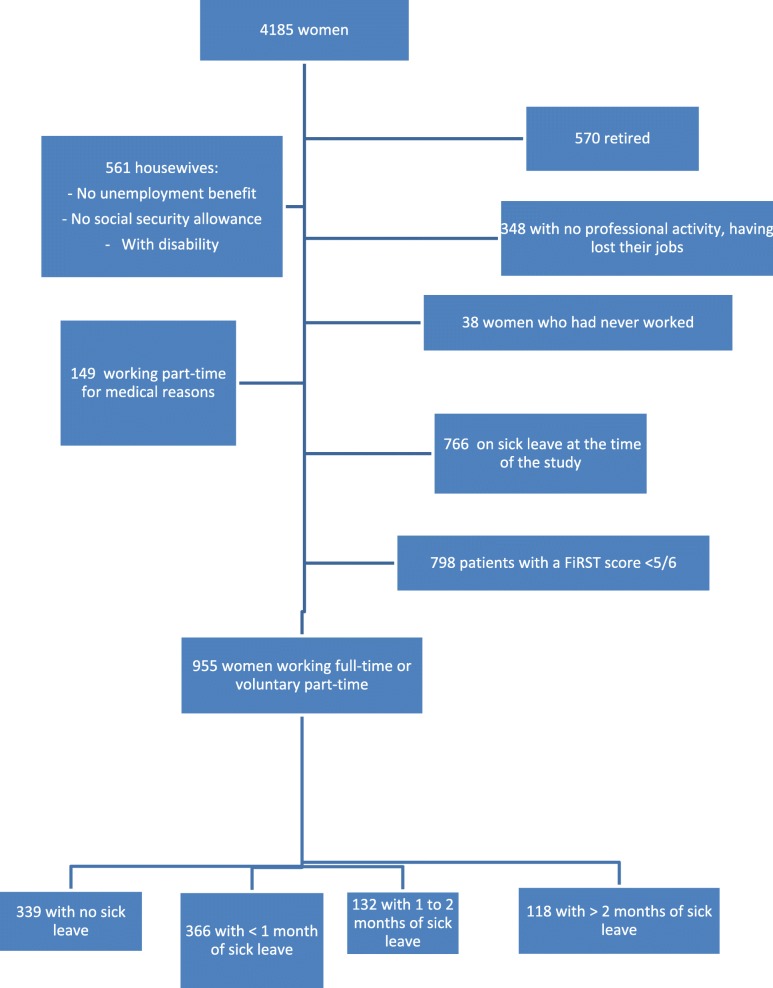
Study flow chart

The population was recruited, via the Internet, on a voluntary basis. The sample included only women of working age (over 18 years of age and not retired), diagnosed with fibromyalgia on the basis of a Fibromyalgia Rapid Screening Tool (FiRST) score of 5/6 or 6/6 [19] and in full-time employment (Fig. 1).

Women who had lost their jobs, were housewives or had never worked were excluded from our study. The sample did not include women in part-time employment for medical reasons at the time of the study. For this last group, in which the women had been allowed to adapt their working time to accommodate their fibromyalgia, we considered the disease to be progressive, preventing these women from resuming their prior professional activities (Fig. 1).

### Data collection

The questionnaire contained 103 questions and took about an hour to complete. It has been described previously [17]. Six domains were explored and the French validated version the FIQ (Fibromyalgia Impact Questionnaire) was also completed [20]. The questionnaire was accessible via the websites of both the french patients’ organizations involved in this study (SOS Fibromyalgia and AFLAR) and via social networks (Facebook). A letter was sent to all the contacts of the patient organizations sponsoring the study. Each included patient received written information about the study. The participant could not responded twice to the questionnaire a “browser fingerprinting” to ensure that. The data were then collected and analyzed by an independent statistician.

### Statistical analyses

Women were grouped according to the duration of sick leave taken during the preceding year. Due to the multimodal distribution of number of days of sick leave preventing analysing sick leave as a continuous variable (Additional file 1: Figure S1), four groups were defined for the analysis, according to population distribution and relevance: women who had taken no sick leave, women who had one to 30 days of sick leave, women who had taken 30 to 60 days of sick leave and women who had taken more than 61 days of sick leave. Univariate comparisons of socio-demographic, clinical and work and workplace factors between the four sick leave groups were performed by using Fisher exact tests and wilcoxon tests as requested. Factors that were associated with sick leave at *p* < 0.20 in univariate analyses were considered to enter into a multivariate polytomous logistic regression model, appropriate for identifying independent risk factors of sick leave categories (the group of women who took no sick leave in the preceding year was considered as the reference group). Results are expressed using the exposure odds ratio (OR), and the 95% confidence interval (CI).

## Results

### Impact of demographic and FM characteristics on sick leave

During the five-month inclusion period, 955 women were included, with a mean of 37 days of sick leave in the preceding year. These women were classified into four groups: no sick leave (36%), up to 1 month (38%), 1 to 2 months (14%), more than 2 months (12%) of sick leave. The women in our study had a mean age of 44 years. Most were living in couples and more than half the women had dependent children. Most had incomes exceeding €1800 per month.

We compared the sociodemographic characteristics of the women between the four groups (Table 1). The women who had taken sick leave at some point in the year were comparable in terms of age, BMI, marital status and income to those who had not taken any sick leave.

**Table 1:** Characteristics of women in full-time work, according to the number of days of sick leave in the preceding year (*n* = 955)

	No sick leave	1 to 30 days of sick leave	31 to 60 days of sick leave	> 60 days of sick leave	*p*
	(*n* = 339)	(*n* = 366)	(*n* = 132)	(*n* = 118)	
	Mean (standard deviation) or *n* (%)	Mean (standard deviation) or *n* (%)	Mean (standard deviation) or *n* (%)	Mean (standard deviation) or *n* (%)	
Sociodemographic Characteristics					
Age	45.3 (8.9)	43.7 (8.9)	45.5 (9.6)	44.6 (9.7)	0.08
BMI	26 (6.1)	26.3 (6.3)	26.7 (5.3)	26.8 (6.2)	0.51
Living with a partner	253 (74.6)	273 (74.6)	90 (68.2)	85 (72)	0.48
Living in an urban area	**166 (49)**	**230 (62.8)**	**88 (66.7)**	**56 (47.5)**	**< 0.0001**
Monthly income					0.33
< €1800	128 (37.8)	123 (33,6)	39 (29.6)	38 (32.2)	
> €1800	211 (62.3)	243 (66.4)	93 (70.45)	80 (67.8)	
Dependent children	**192 (70.6)**	**222 (80.7)**	**82 (77.4)**	**63 (70)**	**0.03**
Clinical Characteristics					
Symptoms:					
Pain	336 (99.1)	362 (98.9)	132 (100)	118 (100)	0.46
Fatigue	336 (99.1)	364 (99.5)	131 (99.2)	117 (99.2)	0.96
Cognitive problems	213 (62.8)	241 (65.9)	97 (73.5)	76 (64.4)	0.18
Sleep disorders	300 (88.5)	342 (93.4)	123 (93.2)	108 (91.5)	0.11
Other health problems	224 (66.1)	250 (68.3)	97 (73.5)	92 (78)	0.07
Associated depression	68 (30.4)	93 (37.1)	34 (35.1)	40 (43.5)	0.14
Associated anxiety	88 (39.3)	124 (49.4)	43 (44,3)	50 (43)	0.12
VAS score for last 48 h	**7.1 (7.1)**	**6.8 (1.9)**	**7 (1.9)**	**7.4 (1.8)**	**0.03**
Impact					
Global FIQ score	54.84 (11.5)	56.92 (8.1)	54.80 (11.6)	57.42 (10.4)	0.41
Regular physical activity	165 (48.7)	162 (44.3)	64 (48.5)	47 (39.8)	0.31
Impact of fibromyalgia on:					
Emotional well-being	314 (92.6)	345 (94.3)	128 (97)	110 (93.2)	0.038
Family life	293 (86.4)	311 (85)	116 (87.9)	102 (86.4)	0.43
Social activities	285 (84.1)	321 (87.7)	121 (91.7)	105 (89)	0.12
Leisure	292 (86.1)	330 (90.2)	122 (92.4)	109 (92.4)	0.13
Marital relations	231 (68.1)	243 (66.4)	88 (66.7)	76 (64.4)	0.81
Feeling of support from partner	**176 (51.9)**	**223 (60.9)**	**76 (57.6)**	**77 (65.3)**	**0.03**
Treatments					
Regular treatment for FM	244 (72)	289 (79)	106 (80.3)	95 (80.5)	0.07
Regular use of painkillers	316 (93.2)	347 (94.8)	124 (93.9)	113 (95.8)	0.71
Use of antidepressants	**251 (74)**	**304 (83.1)**	**112 (84.8)**	**91 (77.1)**	**0.009**
Use of antiepileptic agents	164 (48.4)	182 (49.7)	65 (49.2)	69 (58.5)	0.29

We then compared clinical data between the four groups defined on the basis of the duration of sick leave. We found no significant differences between the groups for pain, cognitive disorders, sleep disorders, fatigue, associated comorbidities, FIQ score, regular treatment or analgesics. The impacts of FM on family life, social activities, hobbies and married life were also similar in the four groups. Two characteristic differing between the four groups were treatment: women who took sick leave were more likely to be prescribed antidepressants than those who did not (*p* = 0.009) and feeling of support from partner (*p* = 0.03) (Table 1).

### Fibromyalgia in the workplace and sick leave

#### Impact of FM on work

Women who had taken sick leave were also more likely to report feeling aggravated by work (84.8, 79.6, 68.6, 62% in women who took > 60 days, 31–60 days, 1–30 days and no sick leave respectively, *p* = 0.0002). More than 68% of the women said that their employers did not recognize their condition, but this lack of recognition was not associated with a longer total duration of sick leave.

Women who had taken sick leave (compared to those who had not taken sick leave) were significantly more likely to report that FM had a significant impact on their working life (96.6, 96.2, 90.7, 86.7% in women who took > 60 days, 31–60 days, 1–30 days and no sick leave respectively, *p* < 0.001) limiting their career progression (*p* = 0.0006), with more work difficulties (*p* < 0.0001) and more stress at work (*p* < 0.0001). The likelihood of reporting these difficulties increased proportionally with the number of days of sick leave taken in the last 12 months (Table 2). Women who had not taken sick leave in the preceding year were more likely to report a lack of support from their coworkers (*p* = 0.05) and their occupational physician (*p* = 0.001) than those who had taken sick leave (Table 2).

**Table 2:** Professional characteristics of the women in full-time work, according to the number of days of sick leave in the preceding year (*n* = 955). Figures are numbers (percentage) unless otherwise stated

	No sick leave	1 to 30 days of sick leave	31 to 60 days of sick leave	> 60 days of sick leave	*P*
	(*n* = 339)	(*n* = 366)	(*n* = 132)	(*n* = 118)	
Commute time (minutes, mean ± standard deviation)	**19.2 ± 22.1**	**23.9 ± 21.7**	**27.2 ± 21.5**	**29.4 ± 26.4**	**< 0.0001**
Stress at work	**225 (66.4)**	**288 (78.7)**	**104 (78.8)**	**104 (88.1)**	**< 0.0001**
Difficulties at work	**239 (70.5)**	**294 (80.3)**	**110 (83.3)**	**105 (89)**	**< 0.0001**
Fibromyalgia preventing career progression	**214 (63.1)**	**252 (68.9)**	**101 (76.5)**	**96 (81.4)**	**0.0006**
Professional adaptation measures in place	**85 (25.1)**	**108 (29.5)**	**56 (40.2)**	**46 (39)**	**0.002**
Transfer to a different post	17 (20)	24 (22.2)	17 (32.1)	13 (28.3)	0.36
Physical adaptation of the post	39 (45.9)	56 (51.9)	27 (50.9)	18 (39.1)	0.49
Adaptation of working hours	32 (37.7)	53 (49.1)	22 (41.5)	20 (43.5)	0.45
Sedentary professional activity	191 (56.3)	216 (59)	74 (56.1)	71 (60.2)	0.81
Prolonged sitting	139 (72.4)	171 (79.2)	62 (83.8)	55 (77.5)	0.19
Prolonged standing	30 (15.6)	34 (15.7)	5 (6.8)	12 (16.9)	0.23
Sedentary activity with repeated movements	**46 (24)**	**55 (25.5)**	**24 (32.4)**	**32 (45.1)**	**0.005**
Manipulation of machines or vibrating tools	15 (4.4)	19 (5.2)	6 (4.6)	11 (9.3)	0.22
Heavy lifting	74 (21.8)	72 (19.7)	22 (16.7)	34 (28.8)	0.10
Thermal nuisance	17 (5)	28 (7.7)	11 (8.3)	12 (10.2)	0.22
Exposure to a noisy working environment	**62 (18.3)**	**96 (26.2)**	**44 (33.3)**	**36 (30.5)**	**0.002**
Working on a computer	**195 (57.5)**	**248 (67.8)**	**92 (69.7)**	**83 (70.3)**	**0.007**
Postural constraints					
Standing position	**156 (46)**	**177 (48.4)**	**60 (45.5)**	**66 (55.9)**	**0.28**
Crouching position	**54 (15.9)**	**54 (14.8)**	**12 (12.9)**	**21 (17.8)**	**0.72**
Arms lifted	**58 (17.1)**	**59 (16.1)**	**27 (20.5)**	**21 (17.8)**	**0.73**
Repeated or high-speed movements	**79 (23.3)**	**84 (23)**	**36 (27.3)**	**31 (26.3)**	**0.70**
Working with one or several joints in a forced position	**107 (31.6)**	**107 (29.2)**	**38 (28.8)**	**41 (34.8)**	**0.65**
Post involving walking	**86 (25.4)**	**120 (32.8)**	**38 (28.8)**	**44 (37.3)**	**0.049**
Impact of work on fibromyalgia					**0.0002**
Improvement	**66 (19.5)**	**57 (15.6)**	**15 (11.4)**	**9 (7.6)**	
Deterioration	**210 (62)**	**251 (68.6)**	**105 (79.6)**	**100 (84.8)**	
No effect	**63 (18.6)**	**58 (15.6)**	**12 (9.1)**	**9 (7.6)**	
Impact of fibromyalgia on professional life	**294 (86.7)**	**332 (90.7)**	**127 (96.2)**	**114 (96.6)**	**0.001**
Feeling of support from work colleagues	**49 (14.45)**	**83 (22.68)**	**26 (19.7)**	**22 (18.6)**	**0.049**
Feeling of support from the occupational therapist	**11 (3.2)**	**25 (6. 8)**	**11 (8.3)**	**16 (13.5)**	**0.001**

#### Job characteristics of the women who did and did not take sick leave

Job adaptation was rare for women with FM, with 69% reporting no adaptation of their working conditions. Recommendations for occupational reclassification and material modifications to and adaptations of working conditions (seemed to be similar in all groups (*p* = 0.36 and *p* = 0.49, respectively), regardless of the number of days of sick leave taken in the preceding year.

Only a small number of specific working conditions were associated with a risk of sick leave: repetitive gestures, noisy workplaces and working with screens were associated with a higher risk of sick leave (*p* = 0.007, respectively). By contrast, women who had and had not taken sick leave in the preceding year were equally likely to have sedentary jobs, with prolonged periods in the sitting position, the carrying of heavy loads, exposure to thermal nuisances and the use of vibrating tools (Table 2).

#### Independent risk factors for sick leave: multivariate analysis

In the multivariate analysis, considering all characteristics associated with sick leave in univariate analyses, to identify independent risk factors of sick leave taken during the preceding year, only the following factors were found to be significantly and independently associated with the risk of sick leave: commute time, work difficulties, limitation of career progression, repetitive gestures at work, and a lack of recognition of the disease by colleagues and bosses (Table 3). Interestingly, all these 5 factors have a “dose effect” relationship with sick leave: the risk associated with each factor increases with duration of sick leave, and most factors were associated with 2.5-fold or more increase in the risk of long sick leave (> 60 days). Commute time and work difficulties were even significantly associated with short sick leave (1–30 days).

**Table 3:** Multivariate analysis of independent risk factors for sick leave (*n* = 955)

	No sick leave Reference	1 to 30 days of sick leave	31 to 60 days of sick leave	> 60 days of sick leave
		OR (95% CI)	OR (95% CI)	OR (95% CI)
Commute time to get to work (+ 10 min^a^)		**1.14 (1.03–1.25)**	**1.17 (1.03–1.31)**	**1.21 (1.07–1.35)**
Difficulties at work		**1.81 (1.13–2.90)**	**3.18 (1.40–7.27)**	**3.21 (1.33–7.75)**
Fibromyalgia hindering career progression		1.46 (0.96–2.22)	**2.17 (1.13–4.15)**	**2.48 (1.23–4.99)**
Sedentary professional activity with repeated movements		1.02 (0.64–1.62)	1.38 (0.74–2.55)	**2.40 (1.31–4.43)**
Difficulty getting colleagues and bosses to recognize the disease		1.45 (0.97–2.17)	**2.80 (1.52–5.16)**	**3.93 (2.00–7.69)**

^a^ Commute time to get to work (+ 10 min^a^) indicates the relative risk with an increase of 10 min in time

Work stress, professional development, sedentary activity, a noisy working environment, working with screens and working shifts were not independent risk factors for sick leave.

## Discussion

In this national internet survey, we found that more than 64% of full-time working women living with FM had taken sick leave during the preceding year, for a total of 37 days on average. Demographic and clinical characteristics were not associated with the risk of sick leave, whereas occupational characteristics were: commute time, difficulties at work, problems with career progression, sedentary position with repetitive gestures and a lack of recognition of FM by colleagues and bosses. These findings are important for the management of FM in the workplace, because most of the risk factors are related to the workplace and can be modified, preferably with the help of an occupational physician aware of the problems to be resolved.

### Sick leave in women with FM

More than two thirds of the professionally active women living with FM included in this study had taken sick leave during the preceding year. This finding is consistent with previous studies, not necessarily restricted to women. In a retrospective Spanish study of 301 FM patients conducted in 2007, 67.8% of the workers questioned had been on sick leave during the year, and the mean number of days off work was estimated at 44 ± 69.6 days [21]. In the United States, according to data for 2009 from the Medical Disability Advisor, the mean duration of sick leave for fibromyalgia syndrome was estimated at 65 days per year [22]. Employees with FM have been found to take three times as much sick leave as other workers without this disease: mean of 29.8 ± 70.6 days of sick leave for subjects with FM, versus 10.4 ± 33.6 days for the total population and 25.7 ± 62.4 days for subjects with osteoarthritis [16]. FM sufferers reported limitations in the performance of their jobs [12, 13, 15], with a significant number of absences for medical reasons [10, 23]. Women who took sick leave were more likely to be prescribed antidepressants than those who did not. The use of antidepressants was clearly declared in the questionnaire by the patients. An explanation could be that’s this prescription was necessary when being at work, and less when being in sick leave. Moreover, women at work or declaring less than 30 days of sick leave, reported better support from their partner. To our knowledge, no data has been published on these 2 observations.

### Impact of FM on the capacity to work, and the role of the occupational physician

Most of the women in our study reported limitations on their capacity to work, with a significant impact on their professional career, difficulties and stress at work. Women with FM often report being less flexible at work, with limitations of their movements and work positions, and difficulties adapting to new and changing work tasks [13]. In a European study published in 2010 (*n* = 299), 74% of employees with FM reported being less productive at work [24]. Physical and mental overload at work can influence the mental and physical symptoms of FM [25], limit the chances of holding down a job [8] and increase stress at work. In a study by Teasell and Merskey, work disability was found to be correlated with the demands of physical work rather than symptom severity [26].

Most of the women had not consulted their occupational physician, but little is known about the role of occupational physicians in helping patients with FM in the workplace. The women who had not been on sick leave in the preceding year were more likely to report a lack of support from coworkers and their occupational physicians than the other women. A lack of understanding and support from coworkers and employers was identified as a factor influencing the ability of subjects with chronic musculoskeletal pain to hold down jobs [27]. A supportive work climate including understanding from colleagues and bosses is important for job satisfaction [13].

### Job characteristics and the risk of sick leave in women with FM

We found that women performing repetitive actions in a sedentary position were more likely to take sick leave. In the Spanish study by Rivera et al. (*n* = 301), sedentary jobs (unspecified) were found to be associated with sick leave (OR: 3.93, 95% CI 1.69–9.13, *p* = 0.001) [21]. In a qualitative Swedish study on FM and employment, the ability of the study subjects to remain in work was found to be related not only to individual work capacity, but also to the work environment and domestic work requirements [13]. Our study showed that difficulties in career progression and getting colleagues and bosses to recognize the disease are associated with sick leave. A japanese study on 15,531 workers followed during 5 years reported reasons for sick leave duration of more than 30 consecutive days [28]. The results showed that workers having support from their employers and colleagues had less days of sick leave. Our study showed also that sedentary professional activity with repeated movements and exposure to a noisy working environment were related to sick leave. The following independent risk factors for sick leave (commute time, difficulties at work, fibromyalgia hindering career progression, sedentary professional activity with repeated movements, difficulty getting colleagues and bosses to recognize the disease) were clinically meaningful. Commute time to get to work and difficulties at work were observed as independent risk factors at the beginning of days of sick leave. Moreover, odds ratio increased with the number of days of sick leave for all the risk factors and were more important after 30 days of sick leave. For example, odds ratio were over 2 in participants having more than 61 days of sick leave.

Other authors reported that job type, uncomfortable working positions with the carrying of heavy loads, repetitive movements, and prolonged sitting or standing have been identified as factors limiting the ability of people with chronic musculoskeletal disorders to hold down a job [27, 29]. In 2005, Henriksson et al. advocated avoiding certain work situations with a heavy physical load, frequent carrying, static or repetitive movements, or movements over the shoulder plane, to improve the ability of women with FM to hold down jobs [5]. In a study by Teasell and Merskey, work disability was found to be correlated with the demands of physical work rather than the severity of FM symptoms [26].

Most of the women in our study had not had any adaptation of their working conditions. The adjustment of work tasks and of the working environment seems to be the main factor influencing the ability of workers with FM to remain in employment [5, 13]. However, it may be difficult to make the necessary adjustments in today’s working world [30].

We found that the duration of sick leave taken by working women with FM increased proportionally with commute time. The journey between home and work should always be taken into account when assessing working capacity, according to Liedberg et al. [13]. These authors pointed out that the ability of individuals to remain in work is dependent not only on their symptoms, but also on the adaptation of the working environment and work tasks on a case-by-case basis [13].

### The clinical and demographic characteristics of FM patients have only a minor effect in the workplace

Our results indicate that demographic and clinical characteristics of FM patients are not risk factors for sick leave. Similarly, Salido et al., in a small study of 51 Spanish women with FM, found no significant relationship between sick leave and sociodemographic characteristics [31]. Indeed, the demographic and clinical characteristics of the women participating in this study were similar to those reported in previous epidemiological surveys [32–35].

Almost all the women in our study complained of pain and fatigue, with more than 90% reporting sleep disorders and over two thirds having cognitive impairment. FM severity, based on FIQ score, was similar in the different groups. In a US cross-sectional study of 2596 people with FM [33], the most commonly reported clinical symptoms were similar to those reported here, including morning stiffness, fatigue, non-restorative sleep, pain and cognitive disorders. The subjects in employment felt that their symptoms compromised their ability to be productive, due to repeated absences and shorter working times [33].

Contrary to our findings, Rivera et al. found, in 2007, that women who stopped work during the year had more clinical manifestations of FM, more associated comorbidities, a worse quality of life and poorer functioning, with a significantly higher total QIF than women who remained professionally active [21]. The number of clinical symptoms (OR = 1.41, 95% CI 1.10–1.82, 0.006) and fatigue (OR 1.07, 95% CI 1.00–1.14, *p* = 0.025) were independently associated with sick leave in this previous study [21].

In our study, women who had not taken sick leave in the previous year were more likely than the others to report a lack of support from their spouse. Liedberg also stressed the vital role of the family circle in keeping people with FM in work, with women stressing the importance of family support to cope with the emotional reactions caused by fatigue and irritation after a day’s work [13].

### Limitations of our study

One of the limitations of this study was the mode of inclusion of the study population, based on self-selection. In addition, only patients with access to the website of the patient organization were able to participate in the study [36]. We did not assess the reason for the sick leave. Moreover, the diagnosis of FM was based on self-evaluation by the women included in the study, and all data were declarative. However, the sociodemographic and professional information for our sample was comparable to that in other published studies.

The cross-sectional analyses presented here only identify associations; they do not establish a causal link. Some key work-related data were missing, as an incident during the online posting of the questionnaire prevented us from obtaining access to some of the data relating to employment.

Moreover many comparisons were performed, resulting in increasing type I error.

## Conclusion

This study of 955 women with fibromyalgia in France is one of the first to describe the socioprofessional and clinical factors associated with sick leave. Our results highlight the predominance of socioprofessional context over clinical and demographic characteristics in the ability of women suffering from fibromyalgia to remain in work. Further studies are required to assess the impact of an early adaptation of working conditions, job description and the development of close professional medical follow-up, on the ability of people suffering from fibromyalgia to remain in employment.

## Supplementary information

**Additional file 1: Figure S1.** Histogram of the number of days of sick leave in the studied sample

## Data Availability

The datasets used and/or analysed during the current study are freely available from the corresponding author on reasonable request.

## Abbreviations

FIQ: Fibromyalgia Impact Questionnaire
FIRST: Fibromyalgia Rapid Screening Tool
FM: Fibromyalgia
OMERACT: Outcome Measures in Rheumatology
