# Supplementary figures and images for: Fibromyalgia in the workplace: risk factors for sick leave are related to professional context rather than fibromyalgia characteristics— a French national survey of 955 patients
Source: BMC Rheumatol. 2019 Oct 26;3:44. doi: 10.1186/s41927-019-0089-0 (PMC6815377; doi:10.1186/s41927-019-0089-0)

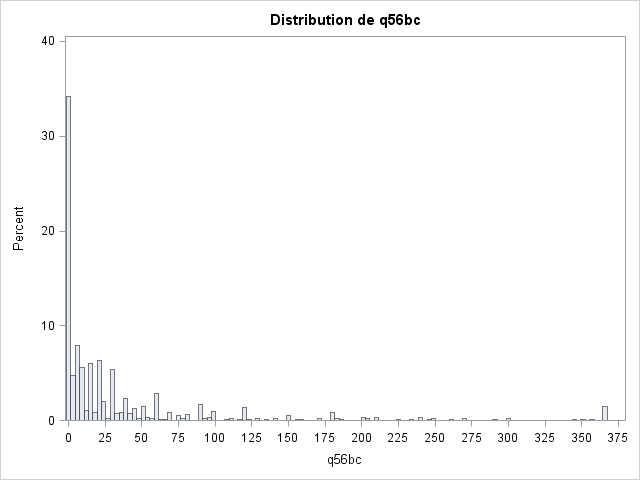


Days

Histogram of the number of days of sick leave in the studied sample

Supplement: Supplementary file 1 — Additional file 1: Figure S1. Histogram of the number of days of sick leave in the studied sample [file 41927_2019_89_MOESM1_ESM.docx]
